# The relation between serum lipids and lutein and zeaxanthin in the serum and retina: results from cross-sectional, case-control and case study designs

**DOI:** 10.1186/1476-511X-11-33

**Published:** 2012-02-29

**Authors:** Lisa M Renzi, Billy R Hammond, Melissa Dengler, Richard Roberts

**Affiliations:** 1 Human Biofactors Laboratory and Vision Sciences Laboratory, The University of Georgia, 601 Psychology Building, Athens, GA 30602-3013, USA; 2 Vision Sciences Laboratory and Human Biofactors Laboratory, The University of Georgia, 520 Psychology Building, Athens, GA 30602-3013, USA; 3 Vision Sciences Laboratory, The University of Georgia, 501 Psychology Building, Athens, GA 30602-3013, USA; 4 Kemin Health, L.C., 600 E. Court Ave, Suite A, DesMoines, IA 50309, USA

**Keywords:** Macular pigment, Lipoproteins, Statins, Lutein, Zeaxanthin

## Abstract

**Background:**

The xanthophyll carotenoids lutein (L) and zeaxanthin (Z) are found in and around the macula of the primate retina, where they are termed macular pigment (MP). Dietary L and Z are absorbed with fat in the gut and transported on lipoproteins to the retina. Both MP and serum lipoproteins have been related to risk for neurodegenerative diseases such as age-related macular degeneration (AMD). L and Z are carried on both HDL (related to reduced risk of AMD) and LDL (related to increased risk). The purpose of this set of studies was to analyze the relation between L and Z in the serum and retina with the circulating lipid profile.

**Methods:**

In all experiments, lipoproteins were measured enzymatically from plasma, and MP optical density (MPOD) was measured using customized heterochromatic flicker photometry. *Experiment 1: Relations between serum L and Z, MPOD and lipoprotein levels*. 108 young, healthy subjects (*M *= 23.2, *SD = *4.12 years) participated. Lipoprotein levels and MPOD were measured. In a subset of 66 participants, serum L and Z levels were also measured using high-performance liquid chromatography. *Experiment 2: Relations between lipoprotein levels and MPOD in statin users*. 20 subjects (*M *= 58.05, *SD = *11.08 years) taking statin medication and 20 subjects (*M *= 57.95, *SD *= 11.03 years) not taking satin were recruited for participation. MPOD and lipoprotein levels were measured. *Experiment 3: lowering lipoprotein levels to impact MPOD*. One individual (aged 41 years) with high MP density adhered first to an atorvastatin regimen, then, after a wash-out period, to a rosuvastatin regimen.

**Results:**

*Experiment 1*: HDL were significantly (*p *< 0.05) related to MPOD (*r *= 0.33), to serum L (*r *= 0.36) and to serum Z (*r *= 0.26). MPOD was also significantly related to total cholesterol (*r *= 0.19). *Experiment 2*: MPOD was not lower in statin users when compared to matched non-statin users, but MPOD decreased significantly with increased duration of statin use (*r *= −0.63). *Experiment 3*: Administration of a statin regimen reduced MPOD with atorvastatin (*p *< 0.05) but not with rosuvastatin.

**Conclusions:**

Serum xanthophylls, retinal xanthophylls and lipoprotein concentrations are significantly related, and changing lipoprotein levels may impact levels of retinal xanthophylls.

## Background

Approximately 600-800 different carotenoids exist [1,2] and serve a multitude of functions such as light harvesting for plant photosynthesis and protection from free-radical damage (for review, see [1-3]). Carotenoids cannot be synthesized by the majority of animal species *de novo *and must, consequently, be consumed in the diet. Of the 600-800 carotenoids that have already been classified in nature, only a small number (about 25) is found in commonly-consumed foods [4] and, therefore, human serum. Carotenoids in the body retain many of their original plant functions, such as light absorption in the eye and in skin, and singlet oxygen quenching in a variety of tissues. Carotenoids can also improve cell membrane stability, alter transcriptional activity, regulate immune function, reduce inflammation and even, possibly, improve neural functioning (e.g., [2,3,5-10]).

The majority of somatic tissues contain carotenoids, and the majority of these tissues contain more than one carotenoid. There are, however, some tissues that tend to absorb one or two carotenoids to the exclusion of others. For example, lutein (L) and zeaxanthin (Z) are found alongside a number of other carotenoids (e.g., alpha-carotene, beta-carotene, beta-cryptoxanthin, lycopene) in tissues such as liver, kidney and lung (e.g., [11]) but are the only carotenoids found in the crystalline lens[12,13] and macular region of the neural retina, where they and their isomer meso-zeaxanthin (MZ) are termed macular pigment (MP) (e.g., [14,15]). In the macula, L and Z are thought to serve many of the above functions, and high MP optical density (MPOD) has been linked to reduced risk of age-related macular degeneration (AMD), an acquired neurodegenerative disease and one of the world's leading causes of blindness [16-20]. AMD has similar risk profiles to other neurodegenerative diseases, such as Alzheimer's disease (e.g., [21-23]), and to cardiovascular disease ([24-29]. Among those risk factors are high plasma concentrations of low-density lipoproteins (LDL) (e.g., [28,30-33], low concentrations of high-density lipoproteins (HDL) (e.g., [30,31,34,35]), elevated plasma triglyceride concentrations (e.g., [29,30,32]), and low serum L and Z levels (e.g., [36-42])

Dietary L and Z are absorbed with fats in the gut and are eventually transported to the liver (e.g., [43]). L and Z within the liver are incorporated into lipoprotein molecules that are transported throughout the bloodstream as part of a carotenoid-lipoprotein complex. The association of individual carotenoids with the various lipid fractions is relatively specific and is determined by the size and hydrophilicity of the carotenoid. For example, pure hydrocarbon carotenoids like beta-carotene and lycopene are carried predominately on LDL fractions, which is largely the basis for the suggestion that they may play a preferential role in reducing LDL oxidation [44]. Other carotenoids, such as the more hydrophilic xanthophylls, show different patterns of transport. L, for example, is carried preferentially on HDL (approx. 52%) and to a lesser extent on LDL (22%) [45]. Past data has shown that circulating levels of L and Z are positively correlated with both serum HDL and LDL fractions but not with VLDL fractions [46].

Given the fact that carotenoids are bound to circulating lipoproteins (and delivered to target tissues via this mechanism), the relation between serum levels of the various lipoprotein and tissue concentrations of carotenoids is of interest. Unlike other carotenoids, retinal L and Z (i.e., MP) can be measured *in situ *and non-invasively using psychophysical methods, such as heterochromatic flicker photometry (HFP) (e.g., [47]). Consequently, it is relatively easy to measure relationships between circulating lipoproteins, serum L and Z levels and MP density. Given the fact that concentrations of both the carotenoid and the lipoprotein carrier are related to risk for AMD (e.g., [16,26,30,31,39]), Alzheimer's disease (e.g., [36,37]) and cardiovascular disease (e.g., [24-28,38,48]), it would be useful to determine whether or not these sources of risk are independent. For example, the question of whether HMG Co-A reductase inhibitors (i.e., statins), increasingly widely used, and which are known to reduce circulating lipoprotein levels, impact the amount of L and Z available to bind to brain and retina has not been studied.

Past research that has addressed the relations between plasma lipoproteins, serum L and Z and MPOD has been conflicting and has largely not addressed the issue of statin use. For example, Broekmans et al [49] originally measured MP using a reflectance method and related those measures to serum carotenoids and plasma lipoprotein concentrations in 376 subjects. Like past studies, they found a positive relation between serum L and plasma HDL and LDL fractions. They also found a negative relation between MP and serum triglycerides but no relation between MPOD and HDL and LDL. These results are interesting but difficult to interpret. For instance, Broekmans et al [49] reported an average serum L and Z level of about 0.23 μmol/L, which is 44% lower than the average normative value reported by the large sample (n = 7059) studied in the Third National Health and Nutrition Examination Survey (NHANES III) (0.41 μmol/L) [50]. Broekmans and colleagues also report lipid profiles that contrast with NHANES III norms (e.g., 23% higher triglyceride levels compared to levels reported by the National Cholesterol Education Program) [51] (16). Approximately 32% of the sample were smokers and, unlike other studies [52,53], MP density was not related to smoking status in their sample. Taken together, the sample and results reported by Broekmans et al. appear to be different enough that their data probably should not be regarded as sufficiently representative to make their results conclusive.

More recently, Loane et al [54] investigated the relationship between serum L and Z, plasma lipoproteins and MPOD. In contrast to Broekmans et al [49], Loane and colleagues found that serum L and Z concentrations were positively related to total cholesterol and HDL, and inversely related to plasma triglyceride concentrations, but not to LDL. No relationships, positive or inverse, were seen between any of the lipoproteins tested and MPOD. Given the conflicting nature of past research in this area, we present a collection of three separate studies, conducted at different times with different samples, that is designed to clarify the relationship between plasma lipoprotein levels, serum L and Z and MPOD in participants varying widely in MPOD and lipoprotein levels (including statin users) across the lifespan.

## Methods

### General

Although each of these experiments was conducted at a different time point, each experiment was approved by the University of Georgia Institutional Review Board, and the tenets of the Declaration of Helsinki were adhered to at all times. All values below are expressed as mean ± standard deviation.

### Participants

#### Experiemnt 1: Relations between serum L and Z, MPOD and lipoprotein levels

108 young, healthy subjects (44 males, 64 females; *M *= 23.20 ± 4.12 years) were recruited from the University of Georgia student research participant pool as part of a larger, ongoing study on the effects of lutein supplementation on visual function. Informed consent was issued and all practices and procedures were approved by the University of Georgia Institutional Review Board. All participants were free of ocular disease, had best corrected visual acuity of at least 20:40 or better (Snellen notation), and reported no history of ocular disease. Participants were given a medical examination by a qualified physician prior to enrollment to ensure general good health.

#### Experiment 2: Relations between lipoprotein levels and MPOD in statin users

A total of 20 individuals who had been taking statin medication for a period of at least four-months prior to enrollment were recruited from the Athens, GA community. These participants (n = 13 males, 7 females, *M *= 58.05 ± 11.08 years) were carefully matched with 20 healthy individuals not on statins (n = 14 males, 6 females, *M *= 57.95 ± 11.03 years) on a number of factors known to influence MPOD, such as BMI, smoking status, presence of Type II diabetes and carotenoid intake. Prospective participants were screened by questionnaire and excluded on the basis of the following parameters: known diagnosis of HIV, use of blood-thinning medications, confirmed diagnosis of sarcoma or carcinoma within the last 5 years, use of chemotherapy and immunosuppressive medications, visual acuity that could not be corrected to 20/60 or better, and failure to meet fasting requirements of 12 hours or more prior to participation.

#### Experiment 3: case-study: lowering lipoprotein levels to impact MPOD

One mildly hyperlipidemic (total cholesterol = 6.18 mmol/L, LDL = 4.15 mmol/L) subject (male, 41 years of age) who was experienced in the psychophysical methodology used in this study (described below) underwent a low-dose (10 mg) atorvastatin (Lipitor®, Pfizer Pharmaceuticals) intervention for one-month. The subject otherwise met inclusion criteria listed for Experiment 2 above and maintained stable dietary and exercise habits during the course of the study. Following a two-year wash out period, the same subject began a one-month regimen (10 mg) of rosuvastatin (Crestor®, AstraZenca Pharmaceuticals). Although statin medications lower cholesterol production via inhibition of HMG-CoA reductase in the liver, lipophilic atorvastatin is, like its counterparts simvastatin and lovastatin, known to cross the blood-brain barrier, unlike hydrophilic rosuvastatin and its counterparts fluvastatin and pravastatin [55]. Both lipophilic and hydrophilic statins were used to ensure that any resulting changes in MP density were due to lipid-lowering activities as opposed to any direct neural influence of statins that cross the blood-brain barrier or, by extension, the blood-retina barrier.

## Materials and methods

### Plasma lipoprotein concentrations

#### Experiment 1

Lipids were analyzed from the plasma samples scheduled for xanthophyll quantitation. LDL, HDL, cholesterol and triglyceride levels were measured in heparinized plasma using commercially available test kits on a Roche-Hitachi Clinical Autoanalyzer. In brief, for LDL and HDL analysis, the respectively bound cholesterol is enzymatically cleaved and oxidized. This oxidation leads to hydrogen peroxide as side-product, which is quantified using a color reaction. For analysis of triglycerides, glycerol is enzymatically freed using lipases, followed by an oxidation leading to hydrogen peroxide as side-product. This is quantified using a color reaction and measured with photometry.

#### Experiments 2 and 3

In order to make lipoprotein analysis more convenient for older adults and for repeated testing on the case study, plasma total cholesterol, HDL, LDL and triglyceride levels were assessed via the Cholestech LDX portable lipid analyzer (Cholestech Corporation). Portable lipid analyzers provide a reasonably accurate and convenient way to screen participants for lipid status [56,57]. The LDX is able to accurately measure lipid profiles when samples are derived from whole blood, when a single, trained operator performs lipid analysis, and when all precautions are taken to avoid error during the collection phase (e.g., swabbing the finger with alcohol, collecting while fingertips are warm, not 'milking' the finger, etc.) [57]. Consequently, all whole blood samples were collected under these conditions. To ensure proper functioning of the LDX system, quality control was performed periodically utilizing known internal standards (Cholestech Corporation).

### Serum L and Z (Experiment 1 only)

Participants in Experiment 1 were, as stated previously, recruited for a larger, ongoing study on lutein supplementation and visual function. Serum was analyzed for L and Z concentration in 66 of those 108 initially recruited participants during phase I of the larger study. For those 66 participants, L and Z levels were determined via high performance liquid chromatography (HPLC), as described previously [58]. Briefly, L and Z were extracted from the aqueous suspension with n-hexane/chloroform. After centrifugation, an aliquot of the organic phase was evaporated to dryness, re-dissolved in mobile phase and analyzed on a normal-phase (Si-60) HPLC system. The detection is performed with a UV/Vis detector at 452 nm for both xanthophylls. The method is a standard one, which is regularly checked for accuracy and precision by attending to inter-laboratory studies organized by the National Institute of Standard & Technologies (NIST) in the USA, and by the Societé Francophone Vitamine & Biofacteurs (SFVB) in the European Union. To assess the daily and long-term laboratory performance of the HPLC plasma analytics, dedicated control plasma is used. The control samples are analyzed at least 4 times/day during the study.

### Dietary assessment (Experiment 2 only)

Fruit and vegetable intake was measured via the commercially available 7-Item Eating Habits Screener^© ^from Block Dietary Data Systems. Burke et al [59] compared the 7-Item Eating Habits Screener^© ^with the more commonly used Fred Hutchinson Cancer Research Center Food Frequency Questionnaire (FFQ) and found that both screeners compared well in their ability to categorize dietary intakes of fruits and vegetables, including L and Z-rich fruits and vegetables, into low, medium, high and very high levels. Scores on both eating habits assessments were also significantly correlated with MPOD, tested at a central retinal locus (*r *= 0.24, *p *= 0.02, n = 96 with the Fred Hutchinson FFQ; *r *= 0.27, *p *= 0.006, n = 96 with the 7-Item Eating Habits Screener^©^).

### MPOD measurement (all experiments)

MPOD was measured psychophysically via customized HFP (cHFP). The method for assessing MPOD via cHFP has been previously described [47,60]. Briefly, the basic measurement procedure involves presenting a small test stimulus that alternates between a measuring wavelength strongly absorbed by MP (460 nm) and a reference wavelength not absorbed by the pigments (550 nm). This stimulus is presented in the center of the fovea and at an eccentric location (7 degrees), which is used as a reference. In effect, the subject perceives a test light that appears to flicker. The subject is then instructed to adjust the intensity of the measuring light until flicker is eliminated. The log difference in the measurement and reference values yields a measure of MP optical density at the test locus. HFP is one of the most widely used and highly validated psychophysical techniques for assessing MP density (e.g., [61]). The denotation of "customized" HFP refers to an ability to optimize viewing conditions (e.g., low-frequency flicker rates) for each subject and has been validated in a wide variety of subjects.

## Results

All statistics were computed utilizing Microcal Origin, version 8.1, and SPSS, version 18.0 (*p *≤ 0.05 defined significance).

### Experiment 1. Relations between serum L and Z, MPOD and lipoprotein levels

Descriptive statistics for all of the major dependent measures are shown in Table 1. A Pearson product-moment correlation matrix was constructed to describe the relation between lipoproteins, serum L and Z, MPOD and BMI (see Table 2). As shown in the table, MP was positively related to HDL (*r *= 0.33, *p *< 0.01) and total cholesterol (*r *= 0.19, *p *< 0.05). MP did not significantly relate to any of the other lipoproteins, or to BMI. Serum L was marginally related to total cholesterol (*r *= 0.21, *p *< 0.09) and was significantly related to HDL (*r *= 0.36, *p *< 0.01). Serum Z was not related to total cholesterol but was significantly related to HDL (*r *= 0.26, *p *< 0.05). These analyses controlled for age and sex.

**Table 1:** Descriptive statistics for participants enrolled in Experiment 1 (n = 108)

Variable	Mean	Standard Deviation	Range	Minimum Value	MaximumValue
Age (years)	23.22	4.11	23.00	18.00	41.00

BMI	23.20	2.67	13.10	19.50	32.60

30-minute MPOD	0.44	0.17	0.89	0.02	0.91

Total cholesterol (mmol/L)	4.42	1.01	5.52	2.77	8.29

HDL (mmol/L)	1.47	0.38	2.05	0.70	2.75

Triglycerides (mmol/L)	1.17	0.68	4.51	0.36	4.87

LDL (mmol/L)	2.43	0.80	4.25	0.98	5.23

Lutein (μmol/L)	0.19	0.10	0.44	0.05	0.49

Zeaxanthin (μmol/L)	0.07	0.03	0.17	0.01	0.18

**Table 2:** Pearson-product moment correlation coefficients between the dependent measures in Experiment 1 (n = 108)

	MPOD	Body Mass Index	Total Cholesterol	HDL	LDL	Triglycerides	Serum L	Serum Z
**MPOD**	**1**	-0.10	0.19*	0.33**	0.11	-0.14	0.31*	0.21†

**Body Mass Index**		**1**	-0.05	-0.00	-0.04	-0.04	-0.07	-0.02

**Total Cholesterol**			**1**	0.42**	0.90**	0.41**	0.21†	0.05

**HDL**				**1**	0.12	-0.20*	0.36**	0.26*

**LDL**					**1**	0.29**	0.10	-0.03

**Triglycerides**						**1**	-0.01	-0.12

**Serum L**							**1**	0.79**

**Serum Z**								**1**

Note: MPOD assessed at 30-min of eccentricity

** = *p *≤ 0.01, two-tailed

* = *p *≤ 0.05, two-tailed

† = *p *≤ 0.09, two-tailed

### Experiment 2. Relations between lipoprotein levels and MPOD in statin users

In order to determine whether statin-users and non-users were well-matched, a one-way ANOVA was conducted on all matching parameters that were on the interval or ratio scales of measurement (see Table 3). No significant differences were seen between statin-users and non-users (*p *≥ 0.05), indicating that the matching procedure was successful. To determine that lipid status was different between statin-users and non-users, a series of independent samples *t-tests *were performed, using the Bonferroni procedure to control for family-wise error. Non-users showed significantly higher total cholesterol (*t *= −3.09, *p *≤ 0.05), LDL cholesterol (*t *= −3.37, *p *≤ 0.05) and LDL:HDL ratios (*t *= −2.05, *p *≤ 0.05) compared with statin-users. Consequently, we can state with some confidence that serum lipid status differed between the two groups, but other potentially confounding factors (e.g., BMI, smoking status, presence of Type II diabetes) were similar.

**Table 3:** Matching parameters for Experiment 2 (n = 40).

Group	Age (years)	Gender	Iris Color*	BMI (kg/m^2^)	Carotenoid Intake Score	Smoking Status	Years Smoking
**Statin** **Users**	58.05 ± 11.08	n = 13 males n = 7 females	2.1 ± 0.9	27.51 ± 5.19	14.1 ± 5.25	n = 13 nonsmokers n = 7 past smokers n = 0 current smokers	5.3 ± 9.18

**Non-Users**	57.95 ± 11.03	n = 14 males n = 6 females	1.9 ± 0.8	26.34 ± 2.73	13.93 ± 3.63	n = 12 nonsmokers n = 4 past smokers n = 4 current smokers	9.10 ± 12.47

Values expressed as mean ± SD unless otherwise indicated

*1 = dark irides, 2 = green/hazel irides, 3 = blue/gray irides

**Figure 1:**
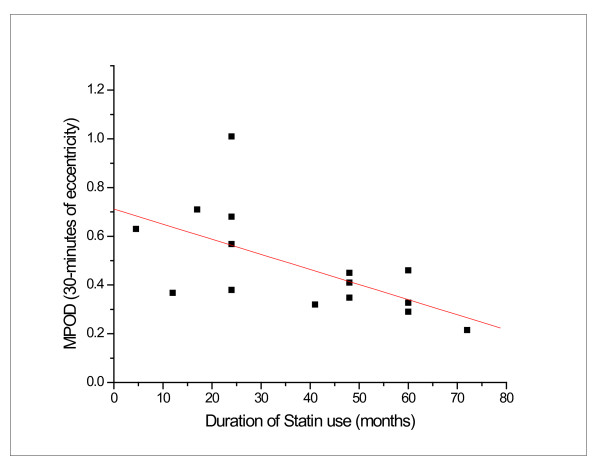
**Decline in MPOD with increased duration of Statin use for participants in Experiment 2; *r *= −0.613, *p *≤ 0.05**. Note that all 20 subjects are represented on the graph, but some points overlap.

A comparison of MP density between statin-users and non-users showed that MP density was not significantly different at any eccentricity along the spatial profile (*p *≥ 0.05). After excluding individuals with Type II diabetes from the analysis, MP density was still not significantly different between statin users and non-users (*p *≥ 0.05). However, a significant inverse correlation between duration of statin use and MP was found at all eccentricities tested (*r *= −0.613, *r *= −0.442, *r *= −0.499, *r *= −0.685 for 10-minutes, 15-minutes, one-degree and two-degrees, respectively) (*p *≤ 0.05), after controlling for age and presence of type II diabetes. An example of one of these relations is shown in Figure 1.

### Experiment 3. Case-study: lowering lipoprotein levels to impact MPOD

**Figure 2:**
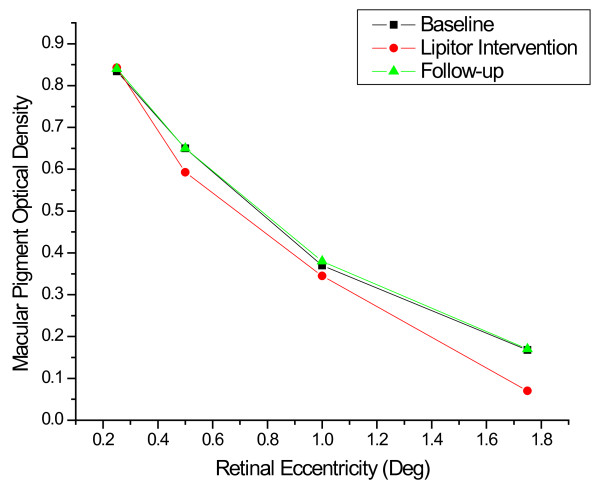
**Spatial profile for MP density in one, well-practiced subject before, during and after intervention with 10 mg atorvastatin**.

Baseline, intervention and post-supplementation averages for the case study participant during both the atorvastatin intervention and the rosuvastatin intervention are shown in Table 4. Differences between MP density and serum lipids were assessed via one-way ANOVA with correction via the Bonferroni procedure to control for multiple comparisons. During the atorvastatin intervention, total cholesterol and LDL cholesterol dropped significantly from baseline (*p *≤ 0.01) and remained low post-intervention (*p *≤ 0.05). In addition, TC:HDL and LDL:HDL ratios were significantly lower during the atorvastatin intervention and post-intervention compared with baseline (*p *≤ 0.05). MP density decreased significantly at the most peripheral locus (1.75-degrees) by about 60% during the intervention (*p *≤ 0.05). MP density rebounded to original levels post-intervention. MP density decreased slightly but not significantly at other loci in the spatial profile during the intervention (See Table 4, Figure 2).

**Table 4:** Baseline, intervention and post-intervention lipid concentrations and MP densities for case study participant

Phase	15-min MP density	30-min MP density	1-deg MP density	1.75-deg MP density	TC (mmol/L)	HDL (mmol/L)	LDL (mmol/L)	TC:HDL (mmol/L)	LDL:HDL (mmol/L)
**AS Baseline**	0.834	0.65	0.37	0.17	5.85 ± 0.33	1.27 ± 0.12	4.15 ± 0.26	4.64	3.29

**AS intervention**	0.843	0.59	0.35	0.07	3.86 ± 0.35	1.39 ± 0.06	2.08 ± 0.45	2.8	1.50

**AS post-intervention**	0.84	0.65	0.38	0.17	4.45 ± 0.25	1.37 ± 0.10	2.65 ± 0.24	3.28	1.94

**Two-year wash-out period**									

**RS Baseline**	0.956	0.728	0.408	0.17	4.71 ± 0.56	1.31 ± 0.12	2.90 ± 0.69	3.62	2.25

**RS intervention**	1.005	0.77	0.485	0.20	3.60 ± 0.31	1.43 ± 0.13	1.91 ± 0.34	2.65	1.35

**RS post-intervention**	1.06	0.72	0.51	0.26	4.95 ± 0.0	1.22 ± 0.0	3.42 ± 0.0	4.1	2.80

AS = atorvastatin; RS = rosuvastatin

Values expressed as means ± SD

*given the fact that there were no significant differences in MP density during the intervention, only one post-intervention was taken

**Figure 3:**
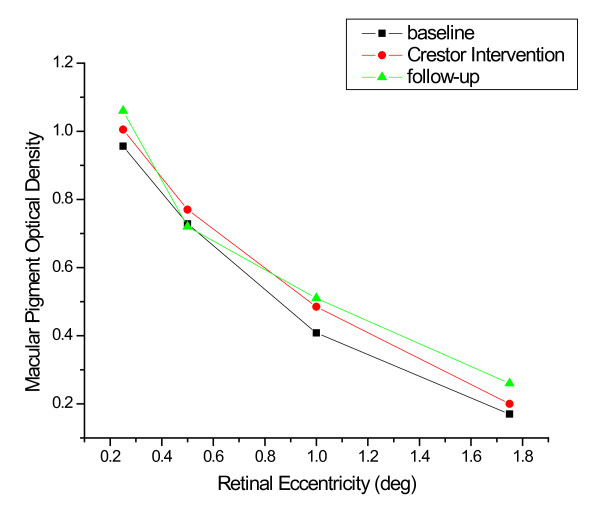
**Spatial profile for MP density in one, well-practiced subject before, during and after intervention with 10 mg rosuvastatin**.

During the rosuvastatin intervention, total cholesterol was significantly lower during the intervention when compared with pre- and post-intervention periods (*p *≤ 0.05). Specific lipid fractions (i.e., HDL and LDL), did not differ significantly when compared independently of the total cholesterol value (*p *≥ 0.05). MP density did not change significantly during the rosuvastatin intervention (See Table 4, Figure 3).

## Discussion

The results of Experiment 1 suggest that MP is related to HDL but not the other lipoprotein fractions. This result is inconsistent with results from both Loane et al [54], who report no relation between MPOD and lipoproteins, and Broekmans et al [49], who report a significant difference between plasma triglycerides and MP. There are some noteworthy differences between these studies that may account for inconsistencies in results. For example, Broekmans and colleagues tested a larger number of subjects (n = 376), and the relation they report is quite small (*r^2 ^*= 0.03). The magnitude of the relationship in our study was similar but the sample was smaller and therefore there was not enough statistical power to reach significance. Also, we screened our sample for conditions that could affect L absorption (e.g., smoking and medical history). Broekmans et al also used different methods to test MP density that could have created a restricted range in their MP data (for review of methodological concerns for MP measurement, see [61]). Hence, it is likely that the differences in study population and methodology account for the differences between our results and those of Broekmans et al. Our results suggest that there is a significant and positive association between serum L and Z, MP and HDL. This finding is consistent with a presumed mechanism of L and Z delivery to the retina. L and Z associate more closely with HDL and Thomson et al. (2002)[62] have suggested that a small proportion (2.5%) of HDL might be responsible for transporting L and Z to the retina (with little or no role for the other lipids). Additional evidence for HDL as the major carrier for L has been provided by Connor et al [63], using WHAM chicks, which are HDL deficient.

The relation between MP and lipid-lowering medications appears to be less straightforward. For example, MP density was not significantly different between statin-users and non-users, despite the fact that lipid levels were higher in non-users. Interestingly, however, when the analysis was confined to statin-users, longer-term statin use was related to reduced MP density. Given the fact that Experiment 2 used a case-control design instead of a longitudinal design, data on MP changes throughout each subject's statin regimen were unavailable. This result is, however, consistent with data from our case-study, in which MP density was assessed throughout the intervention. As shown in Figures 2 and 3, the case study participant showed a reduction in MP density in the more peripheral regions of the retina that was only manifest during the intervention. This effect was only found for the lipophilic atorvastatin and not the hydrophilic rosuvastatin. Of course, the results from one subject are obviously only suggestive and whether the result would generalize is not known. Small effects were noticeable with this particular subject, however, because the subject was extremely well-practiced in the psychophysical task and did not change dietary habits from baseline to intervention within the rosuvastatin and atorvastatin conditions (which were separated by two years). These preliminary data suggest that more research is needed on the question of whether there are differences between rosuvastatin and atorvstatin on retinal health and whether long-term statin-use influences the retinal carotenoids. If stain use does lower MP density over time, it might be advisable to combine medication use with increased intake of carotenoid-rich foods or L and Z supplements. Vishwanathan et al [64] suggest that supplementation with egg yolk, for example, is capable of increasing MPOD in statin users with low MPOD at baseline.

The finding that gross concentrations of serum HDL and total cholesterol are related to MP is important. Poor lipid profiles and low MP levels may pose an increased risk for AMD (as well as other degenerative conditions) (e.g., [16,17,31]). Statins could reduce sclerotic processes within the eye that occur due to high cholesterol levels (e.g., thickening of sclera and Bruch's membrane) and reduce inflammatory processes. L and Z have also been shown to reduce inflammatory processes within the eye [e.g., Izumi-Nagi et al. [7]] and retard atherosclerosis (e.g., Dwyer et al., 2001 [57]). Managing intake of these compounds could help to aggregate their health effects as opposed to one partially offsetting the other.
